# Mucus: An Underestimated Gut Target for Environmental Pollutants and Food Additives

**DOI:** 10.3390/microorganisms6020053

**Published:** 2018-06-15

**Authors:** Kévin Gillois, Mathilde Lévêque, Vassilia Théodorou, Hervé Robert, Muriel Mercier-Bonin

**Affiliations:** Toxalim (Research Centre in Food Toxicology), Université de Toulouse, INRA, ENVT, INP-Purpan, UPS, Toulouse, France; kevin.gillois@inra.fr (K.G.); mathilde.leveque@inra.fr (M.L.); vassilia.theodorou@inra.fr (V.T.); herve.robert@iut-tlse3.fr (H.R.)

**Keywords:** environmental pollutants, food additives, gut barrier, mucus, mucophilic bacteria

## Abstract

Synthetic chemicals (environmental pollutants, food additives) are widely used for many industrial purposes and consumer-related applications, which implies, through manufactured products, diet, and environment, a repeated exposure of the general population with growing concern regarding health disorders. The gastrointestinal tract is the first physical and biological barrier against these compounds, and thus their first target. Mounting evidence indicates that the gut microbiota represents a major player in the toxicity of environmental pollutants and food additives; however, little is known on the toxicological relevance of the mucus/pollutant interplay, even though mucus is increasingly recognized as essential in gut homeostasis. Here, we aimed at describing how environmental pollutants (heavy metals, pesticides, and other persistent organic pollutants) and food additives (emulsifiers, nanomaterials) might interact with mucus and mucus-related microbial species; that is, “mucophilic” bacteria such as mucus degraders. This review highlights that intestinal mucus, either directly or through its crosstalk with the gut microbiota, is a key, yet underestimated gut player that must be considered for better risk assessment and management of environmental pollution.

## 1. Introduction

Synthetic chemicals, including environmental pollutants (heavy metals, pesticides, and persistent organic pollutants) and food additives (emulsifiers, nanomaterials), are widely used for many industrial purposes and consumer-related applications, and there is every indication that such chemicals will become even more abundant in coming decades [1,2,3,4,5,6]. This implies, through manufactured products, diet, and environment, a repeated and growing exposure of the general population to synthetic chemicals.

The gastrointestinal (GI) tract is the first physical and biological barrier against these chemicals, and thus their first target. Their effects on the structure and function of GI compartments through direct and/or indirect exposure, for example, swallowed following mucociliary clearance after inhalation [7], have not been fully investigated. As recently pointed out by Groh et al. [8], an intact intestinal epithelial layer, together with the genera-rich and balanced gut microbiota, is crucial when facing chemical exposure to guarantee a properly functioning gut barrier and the subsequent maintenance of host homeostasis. Indeed, disruption of the gut barrier has been suggested to play a prominent role in the etiology of various noncommunicable diseases, including GI-related irritable bowel syndrome [9], inflammatory bowel disease [10,11], and various other food intolerances and food allergies [12,13], as well as nonalcoholic fatty liver disease [14,15] and autoimmune diseases [16,17,18]. Interestingly, Lerner and Matthias [19] pointed out that the rise in autoimmune diseases throughout Westernized societies has co-occurred with the increasing consumption of food additives in the last decades.

Mounting evidence indicates that various chemicals, including food contaminants and additives, may disrupt the epithelial barrier and/or interfere with the gut microbiota. For a detailed survey of the gut health effects of mycotoxins through the triad of the epithelium/mucus/microbiota, see the review of Robert et al. [20]. In a study in rats, Joly Condette et al. [21] determined the impact of in utero and postnatal chronic daily ingestion of the organophosphate chlorpyrifos (CPF) pesticide on gut maturation. In exposed rats, some alterations in paracellular intestinal permeability, together with changes in tight junction (TJ) gene expression and bacterial translocation, were depicted, depending on the developmental time point: day 21 (weaning) or day 60 (adulthood). The CPF-mediated loss in epithelial barrier integrity was also shown in vitro using differentiated Caco-2/TC7 cells [22]. In another study, Chen et al. [23] investigated in mice the consequences of oral exposure to acrolein, an environmental and dietary pollutant. A loss of barrier integrity and function was depicted with increased permeability and subsequent translocation of bacterial endotoxin-lipopolysaccharide into the blood. Similar results were seen in vitro using Caco-2 cells. Acrolein also caused the downregulation and/or redistribution of three representative TJ proteins (zonula occludens-1, occludin, claudin-1). Interestingly, gut epithelium-targeted alterations have also been described for micron-sized airborne pollutants. In particular, particulate matter (PM, made up of both coarse (PM_10_: diameter < 10 µm) and fine particles (PM_2.5_: diameter < 2 µm), a key pollutant in ambient air, was found to increase gut permeability in mice [24]. In parallel, consequences on epithelial barrier dysfunction have been depicted for nano-sized particles (NPs, <100 nm), mainly in vitro using enterocyte-like cell models for titanium dioxide [25,26], silver [27], or copper oxide [28]. The deleterious impact of single-walled carbon nanotubes has also been demonstrated in vitro [29] and in vivo [30].

As introduced before, the bacteria–xenobiotic crosstalk is increasingly recognized as a key player in gut health. The gut microbiota has the capacity to metabolize different xenobiotics introduced with the diet and to modulate their toxicity to the host [31]; concurrently, these compounds may change the gut microbiota composition and/or metabolic functions, as reviewed for diverse pollutant families, including heavy metals, pesticides, persistent organic pollutants, artificial sweeteners, nanomaterials, and other food additives [32,33,34]. However, the intestinal barrier on its luminal side also involves the mucus layer, which, together with epithelium and microbiota, provides a physical, chemical, and biological line of defense for the host, probably in an orchestrated manner [35,36,37,38,39]. Mucus is a viscoelastic gel that lines and protects the intestinal epithelium, separating it from the lumen contents. It functions as a dynamic barrier that is permeable to gases, water, and nutrients, but impermeable to most microorganisms. The main constituents of mucus are mucins, which are produced, stored, and released by goblet cells. Based on their location and function, several types of goblet cell have been described to date [40]. Interestingly, Birchenough et al. [41] identified “sentinel” goblet cells, which are in charge of protecting colonic crypts from bacterial intruders via mucin secretion from upper crypt goblet cells.

For a long time considered as a “simple” physical barrier, mucus is now recognized to exert other key physiological functions essential for maintaining intestinal homeostasis. In particular, it provides a habitat for the gut microbiota, since the wide diversity of mucin-bound carbohydrates can be utilized by “mucophilic” bacteria as an energy source [42,43,44] or preferential binding sites through bacterial adhesins [45]. Mucus-degrading bacteria possess a large variety of enzymes (glycosidases, sulphatases, sialidases, etc.) to degrade mucus glycans and subsequently harvest oligosaccharides for their own metabolism, and therefore exhibit a colonization advantage competing for the mucosal niche. Numerous mucolytic bacteria have been described to date [46], including *Akkermansia muciniphila* [47], *Bacteroides thetaiotaomicron* [42], *Bacteroides fragilis* [48], *Ruminococcus gnavus*, and *Ruminococcus torques* [49,50]. Some of them have been suggested as biomarkers in gut health and disease [50,51,52]. Another important trait for adaptation of intestinal bacteria to the mucosal environment is their mucus-binding capacity, as shown, for instance, by beneficial lactic acid bacteria. Specific mediators driving adhesion to mucins include pili, such as those produced by *Lactobacillus rhamnosus* GG [53] and mucus-binding proteins, such as in *Lactobacillus reuteri* [54]. Other bacterial cell surface proteins have been identified for promoting reinforced interaction with the host in the intestinal tract [55]. It was also shown that the gut microbiota shapes the colonic mucus phenotype in mice [56]. Depending on the microbiota composition, the penetrability of mucus to bacteria and micron-sized beads was different. Interestingly, high levels of the genus *Desulfovibrio*, belonging to sulfate-reducing bacteria (SRB), were associated with a penetrable mucus phenotype [56].

Until recently, the colonic mucus barrier was described as a “static” two-layered system [57,58,59]. In rodents, colonic mucus was depicted as a loose layer inhabited by bacteria and a layer firmly attached to the epithelium and devoid of bacteria. In mice, the firm and loose mucus layers reached a thickness of ≈50 µm and ≈100 µm, respectively [57] (in rats, thickness of ≈100 µm and ≈700 µm, respectively [59]). In the human colon, a continuous layer (48–273-µm thickness, partly depending on the site) of adherent mucus gel was obtained for freshly resected tissue, with considerable intrasubject and intersubject variability [60]. Later, a 200-μm mucus thickness was reported in human colonic explants [61]. The recent findings of Kamphuis et al. [62] in rodents offered a new view of the structure and function of the colonic mucus barrier. The authors found that the colonic mucus layer covers the faeces instead of the epithelium in the distal colon. This faecal mucus layer confines the microbiota to the faeces and prevents it from remaining in the empty distal colon. In the proximal colon, the mucus does not form a separating layer between the bacteria and epithelium. The mucus/microbiota crosstalk in this revisited system organization should now be further investigated. Intestinal organoids [63,64] could also provide a powerful ex vivo model for mucus/microbiota/synthetic chemical interaction studies.

Concerning the toxicological relevance of the mucus–xenobiotic interplay for the host, little is known about whether—and how—intestinal mucus may influence and/or be modulated, as for the gut microbiota, by environmental chemicals. Based upon this background, the current review aimed to provide a summary of the available evidence regarding the bidirectional relationship between heavy metals, pesticides, persistent organic pollutants, and food additives (emulsifiers, nanomaterials) and the mucus barrier in the gut, also taking into account the consequences on “mucophilic” bacteria.

## 2. Effects of Environmental Pollutants and Food Additives on the Gut Mucus Barrier

### 2.1. Heavy Metals

Inorganic arsenic (iAs) is a ubiquitous environmental contaminant present in its trivalent or pentavalent state in drinking water and some food products [65]. Calatayud et al. [66] studied in vitro the influence of mucus on intracellular accumulation of iAs in the muco-secreting HT29-MTX cells exposed for 24 h to 1 µM As(III) or 10 µM As(V) (Table 1). The percentages of arsenic in mucus were greater (*p* < 0.05) in both cases than those found inside the cells (As(III) 7 ± 3% in mucus vs 1.1 ± 0.3% in cells; As(V) 0.6 ± 0.1% in mucus vs 0.20 ± 0.01% in cells). The authors concluded that the mucus layer impeded iAs cellular uptake and suggested that this retention was responsible for the absence of detection of iAs metabolism in HT29-MTX cells at any of the differentiation stages tested (5, 15, or 21 days post-seeding) (Table 1).

Mercury (Hg) is another heavy metal present in the environment in various chemical forms: metallic mercury, inorganic mercury, and organic mercury. For most of the population, the diet is the main pathway of exposure to this contaminant. Fish and other seafood products mainly contribute to organic mercury in the form of methylmercury (MeHg), while it is accepted that inorganic mercury is the predominant form in foods other than fish and shellfish [67]. Vázquez et al. [68] evaluated in vitro transport and cellular retention of inorganic mercury (Hg(II)) or methylmercury (MeHg) at 3.5 µM, using Caco-2/HT29-MTX cocultures in two proportions (70:30 and 50:50) versus monocultures of Caco-2 cells (Table 1). After 120 min of exposure, for both species, the apparent permeability decreased significantly in the Caco-2/HT29-MTX cocultures with respect to the values found in the Caco-2 monoculture. The increase in the proportion of HT29-MTX cells in the culture also led to an increase in the cellular concentrations of mercury, especially for Hg(II). To support these findings, the authors measured the quantity of mercury present in mucus and found a high accumulation, depending on the mercury species assayed (MeHg: 70%; Hg(II): 30%) (Table 1). They concluded on the protective role of mucus and its trapping properties against mercury species, probably due to their lipophilicity and their high affinity for the mucus cysteine residues.

Cadmium (Cd) is a widespread toxic heavy metal, resulting from both natural and anthropic processes, deriving for the latter from batteries, pigments, plastic stabilizers, pesticides and fertilizers, and photovoltaic devices, as well as from rubber processing, galvanization processes, fossil combustion, and waste incineration [69]. Cd can be released into soil, water, and air, and it bioaccumulates in organic matter by entering the food chain. Grain and cereal products, fish, and offal are the major contributors of Cd exposure in food [70]. Öner et al. [71] evaluated the consequences of oral exposure to high Cd (CdCl_2_ 15 µg/mL in drinking water) for 30 days in rats (Table 1). The authors showed an impairment of the gastric mucosal barrier through a decrease in the mucin content (*p* < 0.01), as revealed by Alcian blue (AB) staining. A negative correlation was found between mucin and cadmium content in the gastric mucosa (Table 1). Later, Asar et al. [72] confirmed that oral exposure to 15 µg/mL Cd in rats for 30 days caused a significant decrease in the mucin content of the gastric mucosa (*p* < 0.01), which was associated to a lower mucus thickness (*p* < 0.01) compared to untreated animals (Table 1). The effects of cadmium administration on mucus have also been depicted for the distal GI regions, such as the colon [73]. The authors investigated the cadmium stress in mice which were supplied with 20 mg/kg (low-concentration Cd) or 100 mg/kg (high-concentration Cd) CdCl_2_ for three weeks (Table 1). For both doses, a significant decrease in the inner mucus layer thickness (*p* < 0.05) was observed in the colon of Cd-treated mice in comparison with controls only treated with water free from CdCl_2_. After the three-week exposure, the populations of lactobacilli and bifidobacteria were significantly decreased (*p* < 0.05) (Table 1). Interestingly, bifidobacteria (*B. longum*) were recently found to restore diet-compromised mucus growth in mice [74].

Lead (Pb) is a highly toxic metal, used in various goods such as tetraethyl lead in gasoline, and highly persistent in the environment over time. Therefore, air, soil, water, and food are all potential exposure routes for Pb uptake. Xia et al. [75] investigated in male adult zebrafish the effects of short-term exposure (seven days) to 10 and 30 µg/L Pb with twice-daily feeding (Table 1). The secretion of mucus, revealed by Alcian blue/periodic acid–Schiff staining (AB-PAS), was increased in the gut of exposed fish (Table 1). The authors concluded that Pb exposure was detrimental to the function of goblet cells and the physical gut barrier. In another study, Wu et al. [76] studied the effects of perinatal Pb exposure on gut microbiota in adult mice, since Pb transfer from mother to offspring occurs transplacentally and lactationally [77]. The Pb-exposed group was supplied with Pb-acetate water (32 µg/mL) and the control group was provided with Pb-free distilled water (Table 1). No direct mucus-related quantification (e.g., mucus thickness, number of goblet cells) was provided. However, among the changes observed in adult gut microbiota in offspring exposed to lead, some specific mucophilic bacterial signatures were depicted in Pb-exposed mice: that is, nearly undetected *A. muciniphila* and enriched *Desulfovibrio* (Table 1).

### 2.2. Pesticides 

Monocrotophos (dimethyl (E)-1-methyl-2-(methylcarbamoyl)vinyl phosphate: MCP) is a broad-spectrum systemic insecticide. Because of its widespread use, MCP has been detected in ground, surface, and rain water [78]. MCP is one of the highest consumed pesticides in India and high levels have been reported in various vegetables such as brinjal, okra, cauliflower, and pea [79]. Vismaya and Rajini [80] investigated the toxic impact of repeated doses of monocrotophos in adult male rats orally treated at different doses (0.45, 0.9, and 1.8 mg MCP/kg body weight (bw)/day) for 30 days (Table 2). MCP affected both the structure and functions of the small intestine. In particular, MCP exposure caused an increase in the number of goblet cells and a goblet cell hypertrophy with cytoplasm vacuolation in the jejunum of rats treated with the intermediate and highest doses tested (Table 2), as shown by scanning electron microscopy and histology.

Chlorpyrifos (O,O-diethyl-*O*-[3,5,6-trichloro-2-pyridinyl]phosphorothioate: CPF) is an organophosphate insecticide used worldwide to treat fruit and vegetable crops. Chlorpyrifos residues are often detected in food and drinking water [81]. Joly Condette et al. [82] studied the effects on pups of oral exposure of female rats to low doses of CPF (1 or 5 mg/kg bw/day vs vehicle controls). Exposure lasted from gestation onset up to weaning of the pups that were individually gavaged (CPF or vehicle) thereafter. Two developmental time points were studied: weaning (day 21: D21) and adulthood (day 60: D60) (Table 2). Perinatal exposure to CPF of 5 mg/kg/day affected intestinal development, together with gut microbiota dysbiosis and increased bacterial translocation. These CPF-associated changes in the gut functioning were more substantial in immature animals (D21), but persisted, albeit to a lesser extent, over time (up to D60). Regarding mucus, impaired *Muc2* gene expression was observed (Table 2).

Imazalil (1-[2-(2,4-dichlorophenyl)-2(2-propenyloxy)ethyl]-1H-imidazole: IMZ) is a widely used pre- and post-harvest fungicide, detected in vegetables and fruits as well as in water and sludge [83,84,85,86]. Jin et al. [87] investigated the consequences of IMZ exposure on male adult zebrafish exposed for 1, 7, and 21 days at IMZ concentrations of 100 and 1000 µg/L (Table 2). The secretion of mucus in the gut, qualitatively characterized with AB-PAS staining, was decreased when the zebrafish were exposed to 1000 µg/L IMZ for 21 days (Table 2). In another study of the same group [88], male mice were orally exposed to chow-mixed IMZ for 28 days at different doses (25, 50, and 100 mg IMZ/kg bw/day) (Table 2). No mucus-related parameters were measured. However, the authors showed an IMZ-induced gut microbiota dysbiosis, accompanied by colonic inflammation for the highest dose tested. In particular, at the genus level, the abundances of *Lactobacillus* and *Bifidobacterium* decreased, while those of *Deltaproteobacteria* and *Desulfovibrio* increased in response to IMZ exposure (Table 2). In a further study, the same group [89] evaluated the effects of IMZ treatment at more environmentally relevant doses (0.1, 0.5, and 2.5 mg IMZ/kg bw/day) on male adult mice for 2, 5, or 15 weeks (Table 2). Significant decreases in the secretion of mucus (*p* < 0.05) were revealed by AB-PAS staining in the colon of mice exposed to 0.1, 0.5, and 2.5 mg/kg bw IMZ for five or 15 weeks, in comparison with control animals (Table 2). The mRNA levels in the colon of *Muc1*/*Muc2/Muc3*, *Klf4*, and *Meprin-β*, encoding proteins involved in mucin synthesis, goblet cell terminal differentiation [90], and detachment and release of mucus from goblet cells [91], respectively; and *Retnlb*, encoding RELM-β, a resistin-like molecule specifically expressed by goblet cells, were then examined (Table 2). The relative mRNA levels of *Muc2* and *Meprin-β* decreased significantly (*p* < 0.05) in the colon after two weeks of exposure to 2.5 mg/kg bw IMZ. When the exposure was extended to five weeks, the transcription levels of *Muc1*, *Muc3*, *Klf4*, *Meprin-β*, and *Retnlb* all tended to decrease, especially in the 2.5 mg/kg bw IMZ-treated group (*p* < 0.05). However, the mRNA levels of *Muc1*, *Muc2*, *Muc3*, *Meprin-β*, and especially *Retnlb* increased significantly (*p* < 0.05) in the colon of mice chronically exposed to a low dose of IMZ for 15 weeks, probably prompting a compensatory phenomenon for mucus layer damage. Changes in *Lactobacillus reuteri* were pointed out [89].

Another example of pesticide-mediated changes in the “mucophilic” population of the gut microbiota has recently been given by Wu et al. [92] in their study on the propamocarb (PM) fungicide, usually used to control a broad spectrum of plant diseases in lettuce, potatoes, and tomatoes [93]. The authors investigated the PM effects on adult mice exposed to 3 (PM 3), 30 (PM 30), and 300 (PM 300) µg/mL PM (corresponding to 0.5, 5, and 50 mg PM/kg bw/day) through drinking water for a duration of 28 days (Table 2). The gut microbiota in the caecum of the PM 300 group deviated from that of the control group. At the genus level, the prevalence of *Oscillospira*, *Parabacteroides*, *Desulfovibrio*, and *Ruminococcus* decreased in the PM 300 group, while the prevalence of *Bacteroides*, *Dehalobacterium*, and *Butyricimonas* increased (Table 2). Interestingly, *Bacteroides plebeius*, which aids in the degradation of dietary polysaccharides, appeared in the PM 300 group [92].

### 2.3. Persistent Organic Pollutants

4-Nitrophenol (PNP) is a persistent organic pollutant isolated from diesel exhaust particles [94]. Tang et al. [95] characterized the effects of PNP in male rats subjected to the following daily oral treatment (200 mg PNP/kg): single dose (one day, 1-d), repeated dose (three consecutive days, 3-d), and repeated dose with recovery (three consecutive days and three recovery days, 6-d) (Table 3). The authors observed that in the 1-d and 3-d PNP treatment groups, the amount of mucus, revealed by PAS staining, increased in the small intestine (duodenum and jejunum), then returned to the basal level in the 6-d PNP treatment group (Table 3).

Benzo[a]pyrene (BaP) is the most characterized and toxic member of the polycyclic aromatic hydrocarbons (PAHs) family. Human contamination to BaP occurs through polluted water and soil exposure, as well as through consumption of food, primarily charcoal-broiled, grilled, and smoked meats and fishes or poorly cleaned vegetables [96]. The inhalation of polluted air [97] or tobacco smoke [96] is another source of BaP exposure. Ribière et al. [98] investigated the effects of BaP on the gut microbiota of male mice orally exposed for 28 days to a BaP dose of 50 mg/kg bw/day (Table 3). BaP exposure induced significant shifts in the composition and relative abundance of stool and mucosa-associated bacterial communities. In particular, shifts in faecal mucophilic bacteria at the end of the experiment were depicted. Among dominant taxa, the relative abundance of Lactobacillaceae (*Lactobacillus*) and Verrucomicrobiaceae (exclusively represented by *A. muciniphila*) decreased. Other bacterial mucus-related taxa, such as *Mucispirillum* and Ruminococcaceae, were also selectively affected (Table 3). Like the dominant members, the rare members of the faecal bacterial communities underwent temporal changes. In particular, an increase in the beneficial genus *Bifidobacterium* was notably observed at the end of BaP oral exposure (Table 3).

Perfluorooctane sulfonate (PFOS) is widely used as surface treatment chemical, polymerization acid, and surfactant in industry, due to its chemical stability, high surface activity, and water- and oil-repelling properties. In 2009, the fourth meeting of the Conference of the Parties to the Stockholm Convention listed PFOS to the Annex B to limit the use of PFOS [99]. Though the application of PFOS has been stopped since 2002, emission persists in many countries due to the lack of cost-efficient alternatives [100,101]. Furthermore, the half-life for clearance of PFOS in the serum is as long as 4.8 years on average [102], meaning that PFOS is bioaccumulative in water and the ground. Suo et al. [103] evaluated the consequences of PFOS exposure using a mouse model of *Citrobacter rodentium* infection (Table 3). Mice were treated daily by oral gavage with PFOS (2 mg/kg) in water for seven days. Control animals received a DMSO vehicle. Mice were then infected with 10^10^ CFU of *C. rodentium*. PFOS treatment continued on a daily basis throughout the whole time course of observation. The authors analyzed the mRNA expression of mucins in colonic tissues. A significant reduction of mRNA expression of *Muc1* and *Muc2* was observed at a late stage (11 days post-infection: p.i.), but not an early stage (five days p.i.) of *C. rodentium* infection in PFOS-treated mice compared to the control, while expression of *Muc3* was comparable between the two groups (Table 3). The mRNA expression of *RELM-β* decreased in PFOS-treated mice at a late but not early phase of *C. rodentium* infection. Consistently, PFOS-treated mice exhibited reduced PAS^+^ area per crypt, indicating reduced mucin or number of goblet cells, compared to the control group at a late but not early stage of *C. rodentium* infection (Table 3). Interestingly, the authors measured the level of mucins and RELM-β under the steady state without infection. Short-term PFOS treatment (11 days) did not affect expression of mucins, but a reduction of *Muc2* was found in mice with longer-term PFOS treatment (17 days) compared to the control, even though no difference was observed in *Muc1*, *Muc3*, or *RELM-β* expression. Furthermore, the authors correlated the combinatorial downregulation of mucins and RELM-β to gut microbiota dysbiosis featured by increased *Escherichia coli* and decreased *Lactobacillus* species, including *Lactobacillus johnsonni* and *Lactobacillus casei*, but not *Lactobacillus acidophilus* and *Lactobacillus reuteri* (Table 3).

### 2.4. Food Additives

Food additives are natural or chemical compounds used by the food industry to increase conservation (antioxidants, preservatives), modify or preserve structural properties (emulsifiers, stabilisers, gelling agents, and thickeners), or improve attractiveness (coloring agents, sweeteners).

#### 2.4.1. Emulsifiers

Emulsifiers are widely used in food products to create interesting textures and taste properties [104]. Oberle et al. [105] investigated the effects of Tween-80, also named Polysorbate-80 (P80, referred as E433), by a single-pass in situ perfusion model in male rats (Table 4). After surgery of jejunum and colon, perfusion with 1% (*w/v*) P80 induced an increase in mucus release, associated with some histological damage, including desquamation of the epithelial surface and necrosis of the mucosal lamina propria. However, after stopping P80 perfusion, mucus release progressively returned to its basal level, showing that the effect of P80 on mucus was reversible (Table 4).

Chassaing et al. [106] investigated in mice the effects of oral chronic exposure (12 weeks) to P80 and carboxymethylcellulose (CMC, referred to as E466), another emulsifier widely used in food products (Table 4). Supplementation with 1% (*v/v*) P80 or 1% (*w/v*) CMC in water or chow led to a decrease in mucus thickness in the colon, as shown by immunostaining. *Muc2* expression remained unchanged. This reduced mucus thickness was associated with substantial changes in the gut microbiota, with notably increased levels of *R. gnavus* and *A. muciniphila* (Table 4). Some bacteria were shown as being in direct contact with epithelial cells. Interestingly, mice treated with P80 or CMC developed a low-grade inflammation associated with a weight gain, adiposity increase, and glucose intolerance. In germ-free mice, 1% (*v/v*) P80 or 1% (*w/v*) CMC water supplementation for 12 weeks did not alter thickness and penetrability of the mucus layer and did not induce any intestinal inflammation, suggesting that emulsifiers are not solely involved in such gut function impairment. To support these findings, microbiota transfer experiments from emulsifier-treated mice to germ-free ones were performed. A decrease in mucus thickness, together with intestinal inflammation and metabolic disorder, was observed in recipient mice, demonstrating the key role of the emulsifier-conditioned gut microbiota on the gut barrier alterations, including mucus.

Induction of gut microbiota dysbiosis with metabolic syndrome and low-grade inflammation in male mice has also been shown with a diet supplemented with 150 mg/kg bw glycerol monolaurate, a natural food emulsifier [107] (Table 4). However, in contrast with the previous study, the authors reported that an eight-week oral exposure to glycerol monolaurate induced a decrease in *A. muciniphila* (Table 4)*.* Another example of emulsifier-mediated “muco-dysbiosis” is given by Shang et al. [108], who investigated in mice the consequences of six-week oral exposure to carrageenan, which is a marine-derived polysaccharide. Thanks to its gelling properties, carrageenan has been used as a thickener and emulsifier in a huge variety of food groups, including low-calorie formulations of beverages, low-fat processed meat, yogurt, infant formula, cheese, jelly, and ice cream [109,110,111]. Mice were given κ-carrageenan, ι-carrageenan, and λ-carrageenan, supplemented in drinking water at a concentration of 20 µg/mL (Table 4). Control animals received normal autoclaved water. Dietary intake of each carrageenan isomer was found to disrupt the epithelial layer and to induce colitis in both proximal and distal colonic regions. The authors also showed that carrageenan modulated gut microbiota at different taxonomic levels; in particular, at the genus level, all isomers of carrageenan significantly decreased the population of *A. muciniphila* (Table 4). Interestingly, *A. muciniphila* was negatively correlated with histological scores of inflammation. 

#### 2.4.2. Nanomaterials

Due to their unique physicochemical properties, nanomaterials, especially NPs, are currently exploited in our everyday environment for a diverse array of products (e.g., textiles, catalysts, detectors, imaging, coatings, packaging, sunscreen, nutraceuticals). Regarding the relationships between NPs and mucus, only therapeutic applications have received sustained attention [112], aimed at designing oral nanomedicines with improved mucopermeability for drug delivery systems. Behrens et al. [113] established in vitro with cells cultured with (HT29-MTX Cl.E12) and without (Caco-2) mucus secretion that mucus acted as a barrier to NPs exhibiting different surface physicochemical properties (hydrophobicity, electronegativity). Bhattacharjee et al. [114] evaluated ex vivo the capacity of functionalized fluorescent silica nanoparticles (SiO_2_-NPs) of different sizes and surface coatings (anionic/cationic) to permeate porcine jejunal mucus. A size- and physicochemistry-dependence in transport was identified, with subsequent effects on mucus viscoelasticity.

In the food industry, NPs are present in food additives/supplements or incorporated into packaging in contact with food, and their use has been projected to increase in the future [115,116]. The presence of NPs in food additives has notably been quantified for titanium dioxide [117,118,119,120,121], silicon dioxide [122,123,124], and silver [125].

Titanium dioxide (TiO_2_, referred to as E171 in Europe) is a white coloring agent widely used in confectionary, soups, beverages, and pastries. A sizable fraction of NPs is produced during the manufacturing process of the powder, accounting for 17–55% of the particles present, depending on the commercial supplier of E171 [120,121,126,127]. Data on interactions between TiO_2_ and mucus are scarce, and until recently, were obtained in vitro with model TiO_2_-NPs in contact with Caco-2/HT29-MTX cocultures (vs Caco-2 monocultures); a facilitated translocation of TiO_2_-NPs in the presence of mucus was reported [26] or not [127], depending on the conditions under study (Table 5). Dorier et al. [127] analyzed, for the first time, food-grade E171 (vs TiO_2_-NPs) in contact with Caco-2/HT29-MTX (70:30) cells, in comparison with Caco-2 cells, during acute (6 h, 24 h, or 48 h, E171 1–200 µg/mL) versus repeated exposure (three weeks, three times a week, E171 1–100 µg/mL) (Table 5). E171 was shown to accumulate more than TiO_2_-NPs under both exposure scenarios (Table 5), due to larger E171 agglomerates, which settled more quickly than their TiO_2_-NPs counterparts did. This resulted in a higher level of cell exposure and cell response, that is, oxidative stress with intracellular ROS accumulation and oxidation of DNA bases. Based on mucus imaging with AB staining, the authors concluded that E171 can cross the mucus layer, since the two cellular models—with and without mucus secretion—accumulated similar amounts of TiO_2_ (Table 5).

Silicon dioxide (SiO_2_, referred to as E551 in Europe) is widely used as an anticaking agent in dried food, cheese, beverages, and confectionary; it has been found to contain nano-sized particles (SiO_2_-NPs) in a range between 5 and 33% [122,123]. The sole study concerning the SiO_2_/mucus interplay has been reported in vitro by Guo et al. [128] for model SiO_2_-NPs. The authors used a Caco-2/HT29-MTX (75:25) coculture, previously characterized as producing a 2–10 μm thick, albeit irregular, mucus layer [129]. Cells were exposed for acute (4 h) or repeated (5 days) periods to amorphous SiO_2_-NPs at three physiologically realistic doses: 0.0002, 0.02, and 2 µg/mL (Table 5). Concerning the barrier function, no mucus-related endpoints were measured, but a significant decrease in the transepithelial electrical resistance (*p* < 0.05) was reported for chronic exposure to low, medium, and high doses of SiO_2_-NPs (Table 5). To date, no study has been reported for the E551 food additive.

Silver (coded as E174 in Europe) is currently used to color the surface of confectionary and pastries. Even though little data is available on the presence of silver NPs (AgNPs) in this food additive [130], a study on silver pearls revealed a subfraction of AgNPs composed of single as well as aggregated/agglomerated particles [125]. Due to their antimicrobial properties, AgNPs are used in plastic food containers and storage boxes, and their potential to migrate to food has been established [131,132,133]. AgNPs have also been proposed as homeopathic remedies and dietary supplements (colloidal silver) for humans and as a feed additive for chicken [134,135] and pigs [136]. The sole in vitro study in the mucus field has been reported by Georgantzopoulou et al. [137] for model AgNPs (10–100 µg/mL) in contact with a Caco-2TC7/HT29-MTX coculture (90:10) under acute exposure conditions (24 h) (Table 5). The mucus layer was able to trap 200-nm AgNPs, thereby reducing their interaction with the cellular membrane and resulting in lower levels of toxicity, oxidative stress, IL-8 release, and proteomic alterations than for 20-nm AgNPs and silver nitrate used as the control (Table 5). The only study in vivo has been performed in male and female rats orally exposed to model AgNPs at a dose of 30, 300, or 1000 mg/kg/day for 28 days [138] (Table 5). Control animals received 0.5% CMC. In the ileum (and also seen in the rectum), a higher number of goblet cells, as revealed by AB-PAS staining, were found to have released their mucus granules, resulting in more mucus in the crypt lumen and ileal lumen (Table 5); in contrast, no significant changes in the goblet cells from the proximal colon were observed for the AgNP-exposed rats. Furthermore, histochemical results showed substantial changes in mucin composition (Table 5), with a decrease in the level of neutral and acidic mucins in goblet cells from the ileum, colon, and rectum mucosa of the AgNPs-treated rats; among the acidic mucin fraction, the proportion of sulfated mucins decreased, while the proportion of sialylated mucins increased. To date, no study has been reported for the E174 food additive.

## 3. Conclusions

**Figure 1:**
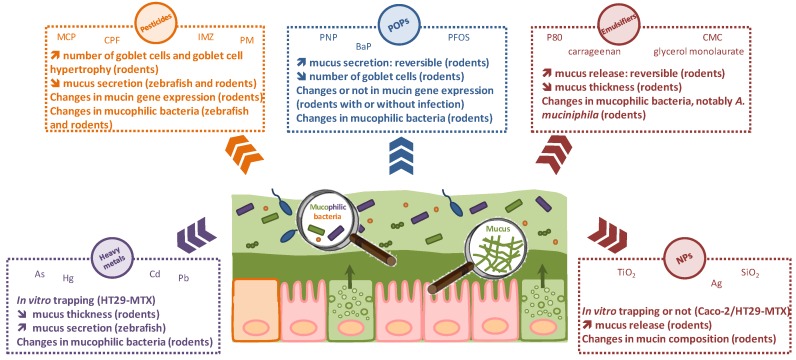
A schematic view of the effects of heavy metals, pesticides, persistent organic pollutants (POPs), emulsifiers, and nanoparticles (NPs) on mucus and mucophilic bacteria in the gut.

The purpose of this review was to highlight the crucial role played by intestinal mucus in the emerging field of gut toxicology. Dietary exposure of humans to environmental pollutants and food additives is of growing concern due to their frequent prevalence in food commodities. The intestinal mucosa is the first physical, chemical, and biological line of defense for the host and thus plays a pivotal role for maintaining gut homeostasis. Mounting evidence indicates that oral exposure to chemicals may disrupt the epithelial barrier and/or interfere with the gut microbiota, with probable consequences in the initiation of inflammatory reactions and clinical manifestations such as intestinal disorders. Although still poorly documented to date, the impact of/on mucus, a key protagonist of the gut barrier, is substantial both in vitro and in vivo for heavy metals, pesticides, persistent organic pollutants, emulsifiers, and nanomaterials, as shown here. A schematic representation is given in Figure 1.

We illustrated the existence of bidirectional relationships between mucus and these various chemical families. Due to its size- and charge-exclusion barrier properties, mucus may trap chemicals, thereby decreasing their toxicity to the host. Conversely, toxicants may affect mucus characteristics (number of goblet cells, thickness and penetrability, mucin expression and composition) all along the GI tract, with potential deleterious effects on the health of the host. The mucus/microbiota interplay and the toxicant-induced “muco-dysbiosis” warrant special attention, since specific mucophilic “biomarkers” of the gut microbiota, for example, *A. muciniphila*, may be highly sensitive to the toxicants via direct and/or indirect mechanisms. Evaluation of an early-life exposure, associated to an immature intestinal barrier, including microbiota, is also of paramount importance. Overall, we conclude that mucus represents a major gut player that must be considered for better risk assessment and management of environmental pollution.

## Figures and Tables

**Table 1:** Effects of heavy metals on mucus and mucophilic bacteria in the gut.

Heavy Metal	Experimental Model	Experimental Design	Main Effects *	References
Arsenic	in vitro HT29-MTX cells	As(III) 1 µM As(V) 10 µM; 5, 15, and 21 days post-seeding; 24 h	iAs accumulation in mucus ↓ arsenic cellular uptake	[66]
Mercury	in vitro Caco-2/HT29-MTX cells vs Caco-2 cells	inorganic mercury (Hg(II)) 3.5 µM, methylmercury (MeHg) 3.5 µM; 2 h	Hg accumulation in mucus ↓ apparent permeability in Caco-2/HT29-MTX	[68]
Cadmium	in vivo Rats	15 µg/mL in drinking water; 30 days	↓ gastric mucin content Negative correlation mucin/cadmium in gastric mucosa	[71]
Cadmium	in vivo Rats	15 µg/mL in drinking water; 30 days	↓ gastric mucin content ↓ gastric mucus thickness	[72]
Cadmium	in vivo Mice	20 and 100 mg/kg; 21 days	↓ colonic mucus thickness ↓ lactobacilli and bifidobacteria	[73]
Lead	in vivo Zebrafish	10 and 30 µg/L; 7 days	↑ mucus secretion	[75]
Lead	in vivo Mice Perinatal exposure	32 µg/mL in drinking water (gestation and lactation)	↓ *A. muciniphila* ↑ *Desulfovibrio* in adult mice	[76]

* ↑ increase and ↓ decrease compared to control conditions.

**Table 2:** Effects of pesticides on mucus and mucophilic bacteria in the gut.

Pesticide	Experimental Model	Experimental Design	Main Effects *	References
Monocrotophos (MCP)	in vivo Mice	0.45, 0.9, and 1.8 mg/kg bw/day; 30 days	↑ number of goblet cells and goblet cell hypertrophy in the jejunum	[80]
Chlorpyrifos (CPF)	in vivo Female rats exposed from gestation to weaning of the pups	1 and 5 mg/kg bw/day; weaning (D21)/adulthood (D60)	↓ *Muc2* expression	[82]
Imazalil (IMZ)	in vivo Zebrafish	100 and 1000 µg/L; 1, 7, and 21 days	↓ mucus secretion	[87]
Imazalil (IMZ)	in vivo Mice	25, 50, and 100 mg/kg bw/day; 28 days	↓ *Bifidobacterium* and *Lactobacillus*↑ *Deltaproteobacteria* and *Desulfovibrio*	[88]
Imazalil (IMZ)	in vivo Mice	0.1, 0.5, and 2.5 mg/kg bw/day; 2, 5, and 15 weeks	↓ colonic mucus secretion ↓ *Muc2* expression (2 weeks) ↑ *Muc2* expression (15 weeks) ↓ *Muc1*, *Muc3* expression (5 weeks) ↑ *Muc1*, *Muc3* expression (15 weeks)	[89]
Propamocarb (PM)	in vivo Mice	3, 30, and 300 µg/mL in drinking water; 28 days	↓ *Oscillospira*, *Parabacteroides*, *Desulfovibrio*, and *Ruminococcus* ↑ *Bacteroides*, *Dehalobacterium*, and *Butyricimonas*	[92]

* ↑ increase and ↓ decrease compared to control conditions.

**Table 3:** Effects of persistent organic pollutants on mucus and mucophilic bacteria in the gut.

Persistent Organic Pollutant	Experimental Model	Experimental Design	Main Effects *	References
4-Nitrophenol (PNP)	in vivo Rats	200 mg/kg single dose (1-d), repeated dose (3-d), repeated dose with recovery (6-d)	↑ mucus secretion in the duodenum and jejunum; reversible	[95]
Benzo[a]pyrene (BaP)	in vivo Mice	50 mg/kg bw/day; 28 days	↓ Lactobacillaceae and Verrucomicrobiacea; changes in *Mucispirillum* and Ruminococcaceae; ↑ *Bifidobacterium*	[98]
Perfluorooctane sulfonate (PFOS)	in vivo Mice	2 mg/kg in drinking water; infection with *Citrobacter rodentium*	↓ *Muc1*, *Muc2*, but not *Muc3* in the colon 11 days p.i. ↓ mucin content in the colon 11 days p.i.; ↑ *Escherichia coli*; ↓ *Lactobacillus*	[103]

* ↑ increase and ↓ decrease compared to control conditions.

**Table 4:** Effects of emulsifiers on mucus and mucophilic bacteria in the gut.

Emulsifier	Experimental Model	Experimental Design	Main Effects *	References
Polysorbate-80	in vivo Rats	1% (*w/v*) single-pass in situ perfusion	↑ mucus secretion; reversible	[105]
Carboxymethylcellulose (CMC)/polysorbate-80	in vivo Mice	1% (*v/v*) P80/ 1% (*w/v*) CMC in drinking water or chow; 12 weeks	↓ colonic mucus thickness; no changes in *Muc2* expression ↑ *R. gnavus* and *A. muciniphila*	[106]
Glycerol monolaurate	in vivo Mice	150 mg/kg bw; 8 weeks	↓ *A. muciniphila*	[107]
Carrageenan	in vivo Mice	20 µg/mL in drinking water; 6 weeks	↓ *A. muciniphila*	[108]

* ↑ increase and ↓ decrease compared to control conditions.

**Table 5:** Effects of nanomaterials on mucus and mucophilic bacteria in the gut.

Nanomaterial	Experimental Model	NPs Size	Experimental Design	Main Effects *	References
Titanium dioxide (TiO_2_) TiO_2_-NPs	in vitro Caco-2/HT29-MTX cells vs Caco-2 cells	12 nm	50 µg/mL; 6, 24, and 48 h	↑ cell uptake in Caco-2/HT29-MTX cells	[26]
TiO_2_ food-grade (E171) and TiO_2_-NPs	in vitro Caco-2/HT29-MTX cells vs Caco-2 cells	E171: 118 nmTiO_2_-NPs: 12 and 24 nm	Acute exposure: 1–200 µg/mL; 6, 24, and 48 hChronic exposure: 1–100 µg/mL; 3 weeks	↑ cell accumulation for E171 vs TiO_2_-NPs; no mucus-mediated trapping	[127]
Silicon dioxide (SiO_2_-NPs)	in vitro Caco-2/HT29-MTX cells	20–30 nm	0.0002, 0.02, and 2 µg/mL; 4 h (acute) and 5 days (repeated)	↓ transepithelial electrical resistance	[128]
Silver (AgNPs)	in vitro Caco-2/HT29-MTX cells vs Caco-2 cells	20 and 200 nm	10–100 µg/mL; 24 h	Mucus trapping for 200-nm AgNPs; ↓ cell-NPs interaction, IL-8 release, and ROS production	[137]
Silver (AgNPs)	in vivo Rats	60 nm	30, 300, and 1000 mg/kg/day; 28 days	↑ mucus release in the ileum and rectum; changes in mucin composition	[138]

* ↑ increase and ↓ decrease compared to control conditions.
